# Tissue-Nonspecific Alkaline Phosphatase in Central Nervous System Health and Disease: A Focus on Brain Microvascular Endothelial Cells

**DOI:** 10.3390/ijms22105257

**Published:** 2021-05-17

**Authors:** Divine C. Nwafor, Allison L. Brichacek, Ahsan Ali, Candice M. Brown

**Affiliations:** 1 Department of Neuroscience, School of Medicine, West Virginia University Health Science Center, Morgantown, WV 26506, USA; dnwafor@mix.wvu.edu (D.C.N.); ali.ahsan95@gmail.com (A.A.); 2 Rockefeller Neuroscience Institute, West Virginia University, Morgantown, WV 26506, USA; 3 Department of Microbiology, Immunology, and Cell Biology, School of Medicine, West Virginia University Health Science Center, Morgantown, WV 26506, USA; alb0037@mix.wvu.edu

**Keywords:** brain microvascular endothelial cells, cerebral microvessels, tissue-nonspecific alkaline phosphatase, *Alpl*, TNAP, blood-brain barrier, stroke, aging, sepsis, Alzheimer’s disease

## Abstract

Tissue-nonspecific alkaline phosphatase (TNAP) is an ectoenzyme bound to the plasma membranes of numerous cells via a glycosylphosphatidylinositol (GPI) moiety. TNAP’s function is well-recognized from earlier studies establishing its important role in bone mineralization. TNAP is also highly expressed in cerebral microvessels; however, its function in brain cerebral microvessels is poorly understood. In recent years, few studies have begun to delineate a role for TNAP in brain microvascular endothelial cells (BMECs)—a key component of cerebral microvessels. This review summarizes important information on the role of BMEC TNAP, and its implication in health and disease. Furthermore, we discuss current models and tools that may assist researchers in elucidating the function of TNAP in BMECs.

## 1. Introduction

Brain microvascular endothelial cells (BMECs) are a key cellular component of the blood-brain barrier (BBB) that provide a stringent, yet, dynamic interface between systemic circulation and the brain parenchyma [1,2]. As opposed to peripheral endothelial cells, BMECs are functionally and morphologically different [3]. For instance, BMECs are characterized by a non-fenestrated continuous endothelium linked together by tight junction (TJ) proteins that confer high resistance to paracellular transport between adjacent endothelial cells [4,5,6]. Likewise, peripheral endothelial cells demonstrate increased transcytosis, whereas BMECs have been shown to exhibit limited transcellular transport [7,8,9]. In addition, BMECs interact with other cell types, such as pericytes, microglia, astrocytes, and neurons to maintain precise cerebral homeostasis [10,11]. The close molecular communication between BMECs and other cell types of the neurovascular unit (i.e., pericytes, glial cells, neurons, and the extracellular matrix) suggest that BMECs may provide paracrine cues that regulate their function in health, and vice versa. For example, earlier studies have revealed that BMECs play a critical role in neurogenesis [12,13], and the interactions between BMECs and the extracellular matrix is also important for the maintenance of the cerebrovascular wall [14,15,16]. Accordingly, injury to this critical interface promotes the infiltration of immune cells, toxins, and proinflammatory cytokines into the brain parenchyma, which, in turn, exacerbates neurovascular unit dysfunction and negatively impacts normal cognitive function [17].

BMEC dysfunction is seen in several neuroinflammatory diseases including sepsis, stroke, multiple sclerosis (MS), and Alzheimer’s disease (AD). Elegant studies have shown that BBB dysfunction, largely mediated through BMECs, precedes cognitive decline or disease activity in normal aging, AD, and MS [18,19,20,21,22,23]. One strategy researchers have taken to mitigate the cognitive decline seen in these neuroinflammatory disease states is to develop therapeutics that target neuronal survival. While this approach has been successful in a number of preclinical studies, clinical trials have been unsuccessful. One potential hypothesis to explain these confounding results is that targeting neuronal survival fails to resolve concurrent BMEC damage, as evidenced by a loss of vascular integrity [24]. Therefore, targeting BMEC dysfunction may resolve neuronal loss. For example, brain-derived neurotrophic factor (BDNF) released from BMECs during ischemic stroke has been shown to protect against neuronal loss [1,25]. Thus, it becomes imperative that therapeutic strategies incorporate the contribution of BMECs or a combination of cerebrovascular and neuroprotective therapies as part of a more comprehensive approach to mitigate brain injury and long-term cognitive impairment seen across many neuroinflammatory diseases.

Several transporters, proteins, and enzymes are expressed on luminal and abluminal surfaces of BMECs and play an important role in regulating the entry and exit of molecules and cells across the BBB [26]. For example, the efflux transporter P-glycoprotein (P-gp) is well-known for limiting central nervous system (CNS) uptake of many small molecules, particularly anti-cancer drugs [27]. Alternatively, the expression of certain BMEC chemokines such as CXCL12 may be redistributed to facilitate the trafficking of leukocytes into the brain during disease [28]. It is also important to note that these proteins/receptors expressed on BMECs may contribute to the regulation of cerebrovascular permeability via distinct downstream pathways. For example, the BMEC tumor necrosis factor-alpha (TNF-α) signaling pathway has been shown to alter expression of the P-gp efflux transporter and junctional protein claudin-5 expression in BMECs [29,30]. Therefore, a functional understanding of the proteins localized to BMECs may be therapeutically beneficial for modulating BBB integrity and permeability. This concept is best illustrated in two recent studies. One study showed that the BMEC major facilitator super family domain containing 2a (Mfsd2a) protein is an important regulator of transcytosis [31], while another separate study showed that BMEC sphingosine-1-phosphate receptor-1 (S1P_1_) is required for size selective entry of small molecules into the brain [32].

Tissue-nonspecific alkaline phosphatase (TNAP) is one of many proteins localized to BMECs, and is highly abundant in human and rodent cerebral microvessels [33]. There are four alkaline phosphatase (AP) isoenzymes in humans and they include: TNAP, germ cell alkaline phosphatase (GCAP), intestinal alkaline phosphatase (IAP), and placental alkaline phosphatase (PLAP). Although TNAP is ubiquitous in many tissue, it is most highly expressed in bone, liver, intestine, kidney, and brain, while the three other AP isoenzymes are expressed in the tissues for which they are named [34]. Despite the high expression of TNAP on cerebral microvessels, the role of TNAP in the brain microvascular endothelium remained unclear until recently [33,35,36,37,38]. In the present review, we discuss current models to study the function of TNAP in microvessels and their challenges. Additionally, we examine emerging data that support a critical role for TNAP in brain microvascular health and disease.

## 2. TNAP Biology

Most of the current knowledge in TNAP biology has been generated from studies on its role in bone and mineral metabolism. This section summarizes the cell biology and gene expression of TNAP, with an emphasis on findings in endothelial cells (ECs) and BMECs.

### 2.1. Cell Biology

TNAP is bound to the plasma membranes of cells via a glycosylphosphatidylinositol (GPI) moiety and functions as an ectoenzyme [39,40,41]. Known substrates for TNAP under healthy conditions include inorganic pyrophosphates (PPi), vitamin B6, adenosine triphosphate (ATP), adenosine diphosphate (ADP), adenosine monophosphate (AMP), and phosphoethanolamine (PEA) [42,43]. For a more in-depth discussion on the structure and substrates of TNAP, the reader is referred to this excellent review [44]. TNAP’s role in bone and mineral metabolism is well-established based on numerous studies showing that diminished TNAP activity causes clinical hypophosphatasia (HPP) in both humans and rodents [45,46,47]. TNAP is also important for appropriate skeletal mineralization. Deficits in TNAP activity result in the accumulation of PPi and osteopontin (OPN), both of which suppress hydroxyapatite crystal growth and formation, i.e., a key inorganic constituent required for bone mineralization [48,49,50]. Furthermore, patients with a clinical hypophosphatasia diagnosis also present with seizures [51]. Subsequent studies have revealed that these seizures are the result of failure to transport vitamin B6 across the BBB. TNAP’s inability to hydrolyze vitamin B6 in the periphery has also been shown to decrease the availability of pyridoxal, a hydrolyzed form of vitamin B6, which is able to cross the BBB. Thus, diminished TNAP activity creates a deficiency of vitamin B6 in the brain, which, in turn, disrupts the synthesis of gamma aminobutyric acid (GABA), an important inhibitory neurotransmitter [52,53]. Despite numerous studies which have demonstrated presumed functions for TNAP in both brain and peripheral tissues, its role in cerebral microvessels remains vastly unexplored.

### 2.2. Global Gene Expression and Enzyme Activity

Although TNAP protein is present in all mammals and in most tissues, its gene (*ALPL* in humans, *Alpl* or *Akp2* in mice) expression varies among different tissues [54]. In the first of its 12 exons, the 5′-untranslated region (UTR) contains either exon 1A or 1B by alternative transcription initiation [55]. Exon 1A is preferentially driven in osteoblasts, while exon 1B is more often initiated by the liver and kidney [56,57,58]. Using marmosets, the TNAP isoform found in brain has been shown to use the same promotor as bone; however, exon 1B is preferentially transcribed by mouse neurons [54]. TNAP activity is also important during mouse embryo development and appears to be the predominant AP seen in 7- to 14-day-old embryos and primordial germ cells [59]. As of May 2021, there are 411 mutations reported in the *ALPL* gene mutations database (http://alplmutationdatabase.hypophosphatasie.com/) (Access on: 13 May 2021), the majority of which are pathogenic loss-of-function mutations that result in HPP. In addition to deficits in bone and teeth development, the depleted TNAP activity in HPP also results in impaired neuronal function that manifests most commonly as seizures [47,51,60]. Conversely, abnormally high levels of TNAP activity in neurons can also result in seizures and neurological dysfunction [60,61,62]. This paradox presents an interesting dynamic where TNAP activity levels must be sustained within a specific range in order to maintain homeostasis within the nervous system.

### 2.3. Gene Expression and Enzyme Activity in BMECs

Two different TNAP isoforms, commonly described as bone TNAP and liver TNAP, are expressed in the brain. The bone-type TNAP transcript is expressed in human and mouse BMECs [54]. During development, TNAP activity in murine cerebral microvessels is delayed and not seen until postnatal day 10. In comparison, TNAP activity has been detected as early as embryonic day 15 in rats [54] and at 28 weeks of gestation in humans [63]. In addition, TNAP enzymatic activity in the brain parenchyma compared to cerebral microvessels differs from one species to another. For example, TNAP activity is weaker in the brain parenchyma compared to cerebral microvessels in humans, monkeys, rodents, guinea pigs, and cats [64,65,66,67]. Conversely, TNAP activity in cerebral microvessels is stronger in the brain parenchyma and weaker in rabbits, frogs, and chickens [64,68] as shown in Table 1. Furthermore, there are significant differences between TNAP activity in cerebral microvessels compared to peripheral microvessels. A comparative study assessing the TNAP activity in BMECs compared to peripheral endothelial cells from the aorta showed that TNAP activity in BMECs is highly elevated compared to peripheral endothelial cells [69]. In addition, Vorbrodt et al. showed that TNAP in the endothelial cells of the liver sinusoids was absent, while skeletal endothelial cells revealed a strong TNAP activity that was discontinuous or irregularly scattered across the plasma membrane compared to BMECs, which showed a continuous and uniform layer of strong TNAP enzyme activity [70]. Given these observations, species-specific and developmental differences in TNAP protein expression and enzyme activity need to be taken into account when elucidating the function of BMEC TNAP in animal models prior to translating these findings to human BMEC TNAP function. Additionally, other cell types that comprise the BBB such as astrocyte end-feet processes and pericytes also exhibit moderate TNAP activity [71,72,73]. Co-culture of BMECs with mixed glial cells/co-cultures *in vitro* has been shown to drastically increase TNAP protein, activity, and mRNA expression in BMECs compared to solo-cultures [74,75,76]. In another study, Tio et al. used a conditioned medium from astrocytes to demonstrate that secreted products from astrocytes were also capable of inducing an increase in TNAP activity in endothelial cells [77].

## 3. Models and Tools to Study TNAP

One of the many challenges to elucidating the role of TNAP in health and disease has been the creation of appropriate genetic and pharmacological tools. This section will address their advantages and disadvantages.

### 3.1. Mouse Models

The life expectancy of mice with a global genetic deletion of the *Alpl* gene (*Alpl*^−/−^) averages 3–5 weeks due to HPP and neurological deficits such as seizures [52,82]. The pathophysiology of HPP is described in a recent review that provides an excellent overview of mouse models where *Alpl* is either genetically or chemically depleted [83]. Due to the side-effects from the HPP phenotype, *Alpl* null mice are inadequate models to study molecular or cellular functions associated with TNAP beyond young adulthood. Alternatively, other researchers have employed adult heterozygous transgenic mice (*Alpl*^+/−^) to explore the consequences of reduced TNAP activity [84,85,86]. Injection of a recombinant mineral-targeted TNAP lentivirus into neonatal mice has been shown to increase AP levels up to 60 days and reduced craniosynostosis [87,88]. Due to the wide and variable expression of TNAP in various cell types, TNAP-expressing cells can also be targeted using the Cre-Lox system. For example, using mice with tamoxifen-inducible inactivation of transforming growth factor β (TGF-β) in TNAP-expressing cells (*Tnap*^cre^;*Tgfßr2*^fl/fl^), researchers have shown that TNAP mitigates TGF-ß-dependent cardiac and skeletal muscle fibrosis through inactivation of SMAD2/3 transcription factors [89].

The majority of research in this field has focused on overexpression of TNAP in peripheral endothelial cells to study vascular calcification. Cell-specific mouse models generated using Cre recombinase transgenic mice and manipulation of the TNAP gene are described in Table 2. Conditional TNAP overexpression has been achieved using an *Hprt*^ALPL^ knock-in mouse, which is described in [90]. Briefly, this model contains a floxed “stop cassette” and human *ALPL* cDNA is inserted into the hypoxanthine phosphoribosyltransferase (*Hprt*) locus on the X chromosome. Cross-breeding of the *Hprt*^ALPL^ with Cre-expressing mice results in deletion of the stop cassette and constitutive *ALPL* expression within the target cell type. Because of the nature of this X-linked system, both homozygous (*Hprt*^ALPL/ALPL^) and heterozygous (*Hprt*^ALPL/−^) females are considered to be over-expressors, however *Hprt*^ALPL/−^ females typically show a milder phenotype. For example, female *TagIn*-Cre^+/−^;*Hprt*^ALPL/−^ mice showed a much milder medial vascular calcification (MVC) phenotype compared to male hemizygous *TagIn*-Cre^+/−^, *Hprt*^ALPL/Y^ mice; however, the effects in female *TagIn*-Cre^+/−^;*Hprt*^ALPL/ALPL^ mice were not described [90].

More recently, *Alpl*^fl/fl^ mice were developed; these mice possess a floxed *Alpl* allele, allowing for cell-specific conditional knock-down of TNAP when crossed with Cre recombinase transgenic mice [94]. Our laboratory has used the *Alpl^fl/fl^* mouse to elucidate the function of TNAP in BMECs. In contrast to the conditional models described previously, we selected a specific *Cdh5*-Cre driver, which codes for VE-cadherin, rather than a *Tie-2-*Cre driver to interrogate TNAP function in these cells. We selected the *Tg(Cdh5-cre)^7Mlia^^A^* strain based on published reports that this specific strain exhibited the highest levels of reporter gene expression in the brain compared to other strains of *Cdh5*-Cre mice [95,96]. By crossing the *Cdh5*-Cre driver mouse with the *Hprt*^ALPL^ mouse described above, our lab also generated a model of TNAP over-expression on endothelial cells (i.e., VE-cOE mice). Using this model, we observed an increase in survival and decreased clinical severity scores at 48 h post-sepsis, indicating to us that TNAP enzyme activity on endothelial cells is important for post-sepsis recovery [35]. More recently, we generated a mouse model with conditional deletion of endothelial TNAP by crossing the *Cdh5*-Cre driver mouse with the *Alpl*^fl/fl^ mouse (i.e., VE-cKO mice). Using primary BMEC cultures from VE-cKO mice, we observed decreased barrier integrity compared to *Alpl*^fl/fl^ controls, further indicating the protective effects that TNAP activity offers to the BBB. These observations have been reported in a preprint [37].

### 3.2. TNAP Pharmacological Tools and Pharmaceuticals

There are several commercially available TNAP inhibitors currently on the market. L-homoarginine (hArg) and levamisole are two of the oldest inhibitors; however, these inhibitors lack selectivity for specific isoenzymes and offer weak binding to TNAP [97,98]. Recent studies have shown that although hArg had no effect on TNAP levels in TNAP-overexpressing mice, it did offer some protection from myocardial remodeling through mechanisms unrelated to TNAP inhibition [92]. Although levamisole has been used in several studies to help identify the role that TNAP plays in health and disease [33,75,99], it is a reversible inhibitor, so cells treated with this drug rapidly regain TNAP activity [100]. In order to identify small molecule inhibitors to target specific AP isoforms, Millan and colleagues employed high-throughput screening approaches and identified several small molecule aryl sulfonamides as potent and selective TNAP inhibitors [99]. The most potent inhibitor identified in this study was 2,5-dimethoxy-N-(quinoline-3-yl) benzenesulfonamide, or MLS-0038949. This molecule is commercially available for purchase and most commonly referred to as TNAP inhibitor (TNAPi); this molecule is also targeted primarily for *in vitro* studies (personal communication, Dr. Jose Luis Millan). Further screening for additional candidates with higher selectivity and potency identified a second inhibitor, SBI-425, which is more appropriate for *in vivo* applications [101]. SBI-425 has been used in several studies related to vascular calcification [94,97,102] and by our own lab to identify its applicability for studying TNAP’s role in the brain. In collaboration with the developers of TNAPi and SBI-425, we demonstrated that the SBI-425 inhibitor is unable to cross the BBB in healthy mice [35]. Since TNAP is localized to both the luminal and abluminal surfaces of brain endothelial cells [70], the inability of SBI-425 to cross the BBB in health may be useful in studying the effects of brain endothelial luminal TNAP activity.

To date, there are few pharmaceuticals available for exogenous treatment to restore AP activity. Enzyme replacement therapy using asfotase alfa (brand name: STRENSIQ) is the first and only, thus-far, FDA-approved medication for patients with perinatal-, infantile-, and juvenile-onset HPP [103,104]. This drug works through the cleavage of inorganic pyrophosphate (PPi) to phosphate (Pi) so that Pi may bind with calcium to form hydroxyapatite crystals needed for healthy bones [48,105,106]. Overall, this treatment has shown to help improve physical function and health-related quality of life in adults with pediatric-onset HPP [102]. Recombinant human TNAP (rhTNAP) purified from rabbit transgenic milk samples and administered to LPS-infected mice resulted in increased survival [107]. Clinical trials using bovine intestinal AP (IAP) have shown functional improvement in patients with sepsis and ulcerative colitis [105,106,108,109]. However, due to side-effects, a human recombinant AP (recAP), which encompasses the stability afforded from placental AP and catalytic viability of IAP, was created and has been promising in clinical trials [110,111]. In preclinical studies recAP did not affect pulmonary inflammation or endothelial and epithelial dysfunction in rats [112]. In a Phase II clinical trial, recAP failed to improve short-term kidney function in patients with sepsis-associated acute kidney injury (sepsis-AKI), although an exploratory finding showed lower mortality in patients who received recAP compared to those who received a placebo [113]. A Phase III clinical trial is now in progress in North America and Europe to test recAP in a larger group of patients with sepsis-AKI.

## 4. TNAP in Brain Microvascular Endothelial Health and Function

TNAP enzyme activity is highly elevated in the BMECs within cerebral microvessels compared to ECs in the peripheral vasculature. Due to this unique, yet poorly understood aspect of TNAP biology, histological detection of AP (i.e., TNAP) activity has been employed as a tool by neuroscientists and anatomists for nearly a century [33]. Please refer to Figure 2 legend for methodology used to quantify TNAP activity. For the remainder of this review, any discussion of TNAP in brain ECs will generally refer to TNAP in BMECs.

### 4.1. TNAP and the Heterogeneity of the Brain’s Vasculature

The brain’s vasculature is heterogenous and displays an arteriovenous hierarchy similar to peripheral vascular beds. Moreover, the expression of a single protein or molecule differs across the cerebral arteriovenous vascular bed. For example, a recent study demonstrated that the expression of von Willebrand Factor (VWF) is increased in venous BMECs compared to brain arterial and capillary BMECs, whereas Mfsd2a protein is highly abundant in capillary BMECs compared to brain arterial and venous BMECs [114]. The localization of TNAP activity in the brain endothelium has been explored in earlier studies. Vorbrodt et al. showed that adult mouse capillary and arterial BMECs exhibit strong TNAP activity, while venules showed a complete lack of TNAP activity. Importantly, despite brain arterioles and capillaries exhibiting strong TNAP activity, electron microscopy showed that capillary TNAP activity is present on the luminal surface, whereas, arterioles demonstrate TNAP activity on the luminal and abluminal surfaces of BMECs [70]. Intriguingly, a recent study utilizing single-cell RNA sequencing demonstrated that TNAP protein, referred to as *Alpl* in this study, is also present in the cerebral venous endothelium. Furthermore, this same study also demonstrated that TNAP protein is highly expressed in brain ECs compared to astrocytes, pericytes, neurons, microglia, and oligodendrocytes [38]. It is unclear whether this new finding contradicts the earlier findings from the Vorbrodt et al. study, which showed an absence of TNAP activity on the venous endothelium using electron microscopy, or whether it simply demonstrates that the protein is present on the venous endothelium but its activity is absent. Regional, or spatial, brain microvascular TNAP activity is also likely to be important. A recent preliminary study from our laboratory showed that TNAP enzymatic activity is higher in the striatum and cortex, compared to hippocampal cerebral microvessels; in TNAP protein localization and/or enzymatic activity throughout brain microvasculature, a mechanistic role for TNAP in the BMECs has also begun to emerge. These results have been reported in a preprint [37].

### 4.2. Potential Mechanism(s) of Action for TNAP in the Brain’s Vasculature

**Figure 1:**
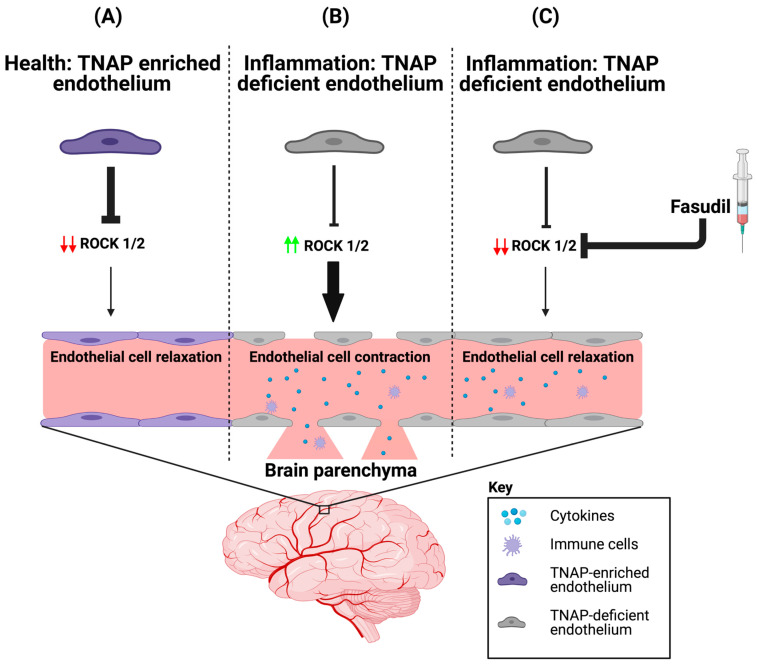
ROCK proteins are involved in BMEC TNAP function. (**A**) In health, BMECs are enriched with TNAP activity. We propose that the abundant TNAP activity decreases ROCK (1/2) protein expression, which maintains the endothelial cytoskeleton by ensuring that brain endothelial cells are in a relaxed state. (**B**) However during inflammation, BMEC TNAP activity decreases substantially, and this decrease promotes increased ROCK (1/2) protein expression (red arrows). Increased ROCK (1/2) protein expression (green arrows) leads to BMEC cytoskeletal contraction and ultimately alters paracellular barrier integrity. Disruption of the BMEC paracellular barrier allows for the infiltration of cytokines and immune cells into the brain parenchyma. (**C**) Therapeutic injection of fasudil (ROCK inhibitor) during inflammation decreases ROCK (1/2) protein expression (red arrows), thereby allowing for a maintenance of paracellular barrier integrity despite the loss of BMEC TNAP activity. BMEC: Brain microvascular endothelial cells; TNAP: Tissue-nonspecific alkaline phosphatase; ROCK: rho-associated protein kinase. Image credit: Biorender.

The molecular mechanisms underlying TNAP function in the brain have been studied more thoroughly in neurons compared to BMECs. For an in-depth discussion of TNAP function in neurons, the reader is directed towards these excellent reviews [53,115,116]. A study from Deracinois et al. was the first to show that inhibition of TNAP using a pan-AP inhibitor (levamisole) in bovine capillary endothelial cells (BCECs) increased cellular barrier permeability to Lucifer Yellow (Pe^LY^) [74]. Barrier integrity can also be quantified using the xCELLigence real time cell analyzer (RTCA) instrument, which measures focal adhesion of cells. By utilizing this barrier assay, recent data from our laboratory demonstrated that pharmacological inhibition of TNAP by the previously described highly specific TNAP inhibitor, TNAPi (see Section 3.2), in a human BBB endothelial cell line (hCMEC/D3 cells), increased paracellular barrier permeability. We have also shown that primary BMECs (pBMECs) cultured from adult mice with a conditional deletion of endothelial TNAP (VE-cKO mice, see Table 2) display diminished paracellular barrier permeability. Next, we demonstrated that loss of TNAP induced cytoskeletal remodeling, as quantified by phalloidin and vimentin immunofluorescence, via the increased BMEC expression of rho-associated protein kinase (ROCK) 1 and 2 proteins by using in-cell westerns (ICW). Lastly, we showed that fasudil, a pan-ROCK inhibitor, mitigated the worsened paracellular barrier integrity seen in TNAPi-treated hCMEC/D3 cells or VE-cKO pBMECs. These collective preliminary results have been reported in a preprint [37]. Taken together, our results demonstrate a novel mechanism through which TNAP is able to maintain barrier integrity in brain endothelial cells (Figure 1) and may explain why drugs like fasudil have shown some therapeutic efficacy in preclinical models of sepsis and stroke [117,118,119,120]. However, there are some potential limitations to this proposed mechanism. For example, the Deracinois et al. study showed that BCECs co-cultured with mixed glial cells did not exhibit an increase permeability to Pe^LY^ following levamisole treatment [74]. Consequently, it is unclear how this new mechanistic pathway may operate *in vivo* in the presence of other cell types found in the BBB. Future studies will need to address such limitations *in vivo* to understand how cell-cell interactions within the BBB contribute to TNAP function in BMECs.

Several molecules such as retinol, cyclic AMP (cAMP), glucocorticoids, transforming growth factor-beta (TGF-β), interleukin-6 (IL-6), and basic fibroblast growth factor (bFGF) are known to modulate TNAP activity [33]. While retinol, cyclic cAMP, glucocorticoids, IL-6, and bFGF have been shown in previous studies to increase TNAP activity [115,116,121,122], TGF-β has been shown to suppress TNAP activity in brain ECs [122]. This non-exhaustive list of molecules highlights their potential importance in regulating TNAP function in brain ECs; however, the mechanisms through which these molecules modulate TNAP activity remain to be elucidated. Likewise, it remains unclear how the presence of TNAP activity on brain ECs influences targeted downstream pathways associated with these molecules.

## 5. A Role for Endothelial TNAP in the Brain’s Microvasculature in Aging and Disease

A significant number of studies have demonstrated a role for peripheral endothelial TNAP in disease [92,97,102,123]; however, there is a paucity of data on the function of brain microvascular TNAP activity in disease states. In this section, we examine emerging data for the role of brain microvascular TNAP in aging and disease.

### 5.1. Brain Microvascular TNAP in Disease

BBB dysfunction is a common neuropathological feature seen across many neuroinflammatory diseases [17]. Yet, there are no specific therapies that effectively mitigate BBB dysfunction to treat neurological disorders. Historically, quantification of TNAP activity has been used as a histological marker to detect changes in cerebral microvessel density or morphology in neurological disease. In spite of widespread TNAP activity as a histological tool, there was a minimal emphasis on physiological explanation regarding why the loss of TNAP activity occurred and how the loss of TNAP activity might affect disease progression [33]. This perspective is exemplified in an earlier study that quantified TNAP activity in cerebral microvessels to demonstrate that patients with leukoaraiosis (LA) exhibited decreased vascular density [124]. Yet, it was not evident from this study why TNAP activity was decreased in patients with LA compared to age-matched controls. It was also unclear if the loss of TNAP activity in LA was due to the loss of cerebral microvessels since the study did not utilize appropriate markers for the vasculature such as CD31, CD34, or collagen type IV [125]. Owing to these findings, our laboratory identified several important questions for future investigations: (1) Is the loss of TNAP activity on cerebral microvessels specific to a unitary disease state; (2) Does the loss of TNAP activity equate to the loss of cerebral microvessels; (3) Does diminished TNAP activity in cerebral microvessels result in alterations of BBB integrity; and (4) What are the relevant long-term neuroinflammatory and behavioral implications of the loss of TNAP activity in BMECs? Recent studies from our laboratory have begun to answer these new questions.

To elucidate whether BMEC TNAP activity was altered in systemic inflammation, we utilized the cecal ligation and puncture (CLP) model of sepsis to determine whether TNAP activity changed post-sepsis, and if early changes in TNAP activity were sustained long-term, i.e., seven days, into late sepsis. Our results showed that loss of TNAP activity is present as early as 24 h post-sepsis [10] and this loss was sustained up to seven days post-sepsis [36]. However, it was unclear whether the resultant loss of TNAP activity was due to cerebral microvessel loss as postulated in earlier studies [124]. Therefore, we combined CD31-immunolabelling of cerebral microvessels with histological detection of TNAP activity. Our results showed that the loss of TNAP activity was not due to a loss of cerebral microvessels, as the density of CD31+ cerebral microvessels remained consistent while TNAP activity decreased in the presence of septic injury. This important finding refutes the utility of brain microvascular TNAP activity as a measure of cerebral microvascular density since this activity changes with disease. More importantly, we showed that the loss of TNAP activity on cerebral microvessels coupled the loss of barrier integrity through the loss of junctional protein claudin-5. We also demonstrated that loss of TNAP activity was associated with increased microgliosis, astrocyte proliferation, and the infiltration of an endogenous permeability marker, immunoglobulin G (IgG), into the brain of septic mice [36]. To further demonstrate the role of brain microvascular TNAP in disease, we intraperitoneally injected septic mice with vehicle or SBI-425, an *in vivo* TNAP inhibitor discussed in Section 3.2, and subsequently examined claudin-5 expression. Our results showed that claudin-5 expression was decreased in SBI-425-treated septic mice compared to vehicle-treated septic mice. Since sepsis survivors are burdened with long-term cognitive impairments [126], we also examined whether the loss of TNAP in cerebral microvessels was coupled to behavioral deficits at seven days post-sepsis. Our results demonstrated that septic mice displayed an impaired sensorimotor phenotype but had no memory deficits. We speculate that the impairment in sensorimotor dysfunction most likely resulted from the observed loss of TNAP in the cortex, striatum, and spinal cord. In contrast, we attribute the lack of observed memory deficits to the absence of any changes between sham and septic mice when hippocampal TNAP activity was quantified [36]. Nevertheless, it remains unclear whether hippocampal TNAP activity is decreased at later timepoints (>seven days post-sepsis) when memory loss has been observed in other studies in septic mice [127,128].

**Figure 2:**
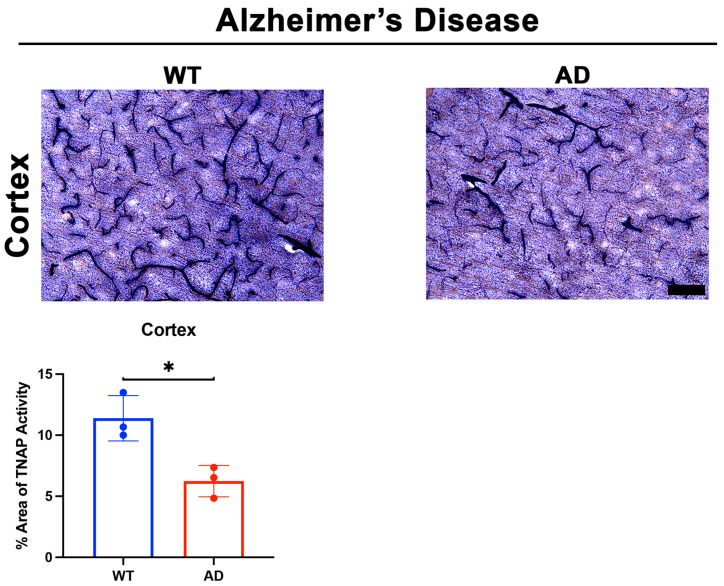
TNAP activity in a mouse model of AD. Preliminary data from male CVN-AD (AD) mice demonstrate a significant decrease (*p* = 0.01) in brain microvascular TNAP activity in the cortex compared to WT (C57BL/6J) mice. Data were analyzed using unpaired student’s *t*-test and *n* = 3/group. TNAP activity tissue histology was performed as previously described [36]. Briefly, tissue sections were evaluated for alkaline phosphatase activity using the BCIP/NBT substrate kit (SK-5400, Vector Laboratories, Burlingame, CA) according to the manufacturer’s instructions. BCIP/NBT is an AP substrate that undergoes an oxidation/reduction reaction when dephosphorylated by AP. The result of this dephosphorylation is visualized as a blue color change at the site of the reaction. This substrate can be used to measure AP activity in tissue sections or cells. Enzyme activity in sections is quantified using ImageJ. TNAP: Tissue-nonspecific alkaline phosphatase; AD: Alzheimer’s disease; WT: Wild-type. Images taken at 20× magnification and scale bar = 75 µm. * indicates *p* < 0.05.

Two important questions emerged after these intriguing findings: (1) What molecules initiated the loss of BMEC TNAP activity in sepsis, and (2) Was the loss of TNAP activity unique to sepsis or was it present in acute neuroinflammatory conditions with BBB disruption such as stroke? Indeed, our laboratory recently reported in a preprint that the loss of TNAP activity in sepsis manifests similarly in the cortical and striatal penumbra following 60 min transient middle cerebral artery occlusion, a model of experimental ischemic stroke, in mice [37]. Since animal models of sepsis and stroke have been shown to exhibit a rapid increase in systemic and cerebral pro-inflammatory cytokines [123,129,130,131], we hypothesized that the presence of pro-inflammatory cytokines would decrease BMEC TNAP activity. As expected, treatment of hCMEC/D3 cells with TNF-α and interferon-gamma (IFN-γ) significantly decreased BMEC TNAP activity, thereby resulting in both the loss of barrier integrity similarly to what was observed when BMECs were treated with TNAPi alone [37] (results reported in a preprint). Taken together, these novel findings have begun to reveal a functional understanding of brain microvascular TNAP function in acute disease conditions. Conversely, a comparable understanding brain microvascular TNAP’s function in chronic neuroinflammatory disease conditions such as AD remains to be elucidated. We present here for the first time preliminary evidence suggesting that brain microvascular TNAP activity is decreased in a model of AD (APPSwDI/Nos2^−/−^ or CVN-AD [132]) compared to wild-type (WT) mice (Figure 2). The CVN-AD mouse exhibits AD-like pathologies and behavioral outcomes such as parenchymal beta-amyloid deposition, vascular amyloid angiopathy, neuronal loss, and tau hyperphosphorylation independent of a tau mutation. Additionally, the mouse also exhibits cognitive and sensorimotor deficits along with metabolic and sleep disturbances, all of which have been characterized in AD patients [133,134]. This result contrasts with earlier studies, which demonstrated that elevated neuronal TNAP activity exacerbates tau toxicity through the dephosphorylation and subsequent destabilization of extracellular tau released into the brain parenchyma from dying neurons [135]. Further studies are necessary to dissect the role(s) of both brain microvascular TNAP and neuronal TNAP in AD pathogenesis.

### 5.2. Brain Microvascular TNAP in Aging

**Figure 3:**
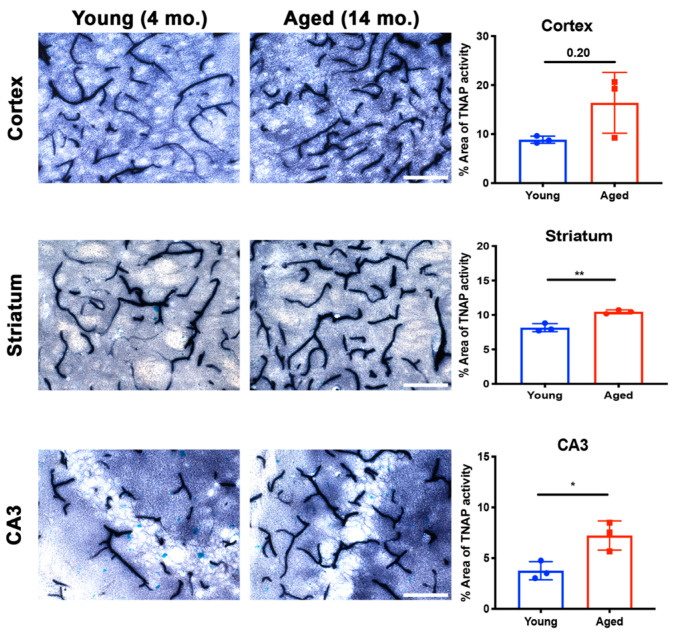
Brain microvascular TNAP activity in aging. The striatum (*p* = 0.004) and hippocampal CA3 (*p* = 0.02) regions revealed a significant increase in brain microvascular TNAP activity in aged male mice (14-month old) compared to young male mice (4-month old). The cortex also revealed an increase in TNAP activity in aged mice; however, this increase was not statistically significant (*p* = 0.20). Data were analyzed using unpaired Student’s *t*-test with *n* = 3/group. Histological staining to quantify TNAP activity was performed as previously described [36]; also see Figure 2 for a detailed description of this method. TNAP: Tissue-nonspecific alkaline phosphatase. Images taken at 40× magnification and scale bar = 100 µm. * indicates *p* < 0.05 and ** *p* < 0.01.

Aging increases susceptibility of brain ECs to injury or toxins [136]. Preclinical studies utilizing plasma substitution therapies from aged mice into young mice have elucidated the impact of aging on brain EC function and zonation of the cerebrovasculature [137,138]. While a clear role for BMEC TNAP in aging is not well- understood, recent findings suggest that brain microvascular TNAP protein, referred to as *Alpl*, is decreased in the hippocampus of aged mice (20 months old) compared to young mice (3 months) [138]. Given the observation of increased TNAP protein in aging, we examined whether brain microvascular TNAP activity is also increased with aging. As shown in Figure 3, we found that TNAP activity is increased in cerebral microvessels of the cortex, striatum, and hippocampus (CA3 shown) in 14-month-old mice compared to 4-month-old mice. These results suggest a dichotomy in brain microvascular TNAP function whereby TNAP activity is decreased in acute injuries like sepsis and ischemic stroke, while TNAP activity is increased in aging. The implications of the increased brain microvascular TNAP protein were further explored in a new report, which showed that intraperitoneal injection of TNAPi, i.e., MLS-0038949, in aged mice enhanced transcytosis of plasma proteins into the brain parenchyma [38]. To our knowledge, these are the only studies that have investigated a functional role for brain microvascular TNAP in aging. Importantly, transcriptomic profiling has shown that vessel segments, i.e., arteries, capillaries, or venules, exhibit different changes in their transcriptomic profiles during aging [138]. Overall, these findings emphasize the need to consider the impact of zonation, differential functions of the vascular tree, and as well as regional differences when studying BMEC TNAP function in normal health and in disease.

## 6. Conclusions

The goal of this review was to summarize the current knowledge on TNAP function in BMECs, and by extension, the BBB. New TNAP mouse models and pharmacological tools have enabled researchers to uncover novel insights on TNAP function in BMECs. In this review, we synthesized the current literature on brain microvascular TNAP as well as provided preliminary evidence that demonstrates a role for brain microvascular TNAP in aging and AD. Future investigations will focus on a rigorous validation and extension of these findings. Ultimately, a better understanding of TNAP function in normal health and in disease will shift the neuroscientist’s view of this enzyme from a histological marker of cerebral microvessels to a major player in cerebrovascular health and homeostasis.

## Figures and Tables

**Table 1:** Species-specific TNAP activity in the CNS.

Animal Models	TNAP Activity in Cerebral Microvessels	Gene Name	Time Period When First Detected in CNS	Ref.
Zebrafish	Activity is present in the brain parenchyma and vessels	*alpl*	11 h post-fertiilzation (hpf)	[78]
Mouse	Strong activity in vessels and weaker activity in the brain parenchyma	*Alpl* or *Akp2*	Embryonic day 7–14	[59,64]
Rat	Strong activity in vessels and weaker activity in the brain parenchyma	*Alpl* or *Akp2*	Embryonic day 15	[54,64]
Guinea Pig	Strong activity in vessels and weaker activity in the brain parenchyma	*Alpl*	-	[64]
Frog	Activity is absent on vessels but present on inner arachnoid and perineurium	*alpl*	-	[68]
Chicken	Weak activity in vessels and strong activity in the brain parenchyma	*ALPL*	Day 2 of the incubation period	[64,79]
Rabbit	Activity is absent in vessels but strong in brain parenchyma	*ALPL*	-	[64]
Cat	Strong activity in vessels and weaker activity in the brain parenchyma	*ALPL*	-	[64]
Rhesus Monkey	Strong activity in vessels and laminar activity pattern in the brain parenchyma (i.e., some areas/layers have higher activity than others)	*ALPL*	-	[80]
Human	Strong activity in vessels and laminar activity pattern in the brain parenchyma (i.e., some areas/layers have higher activity than others)	*ALPL*	28 weeks of gestation	[63,81]

**Table 2:** Cell-Specific *In Vivo* Mouse Models for TNAP.

Mouse Model Type	Cre-Mediated Target Cell	Preclinical Outcomes	Ref.
TNAP OE	Vascular smooth muscle cells (*Tagln*-Cre or *SM22*-Cre)	Mice had increased AP enzyme activity, increased systolic blood pressure, and increased vascular calcification. Male TNAP-OE mice lifespan was shorter than WT controls, as all died by 5 months of age.	[90]
TNAP OE	Endothelial cells (*Tie2*-Cre)	Increased AP activity was localized to the luminal side of the aorta and vascular networks of heart, lung, kidney, liver, small intestine, and pancreas. No skeletal abnormalities were detected; however, in the heart, kidney, mesentery, pancreas, spleen, and lung parenchyma there were calcified lesions in adult mice.	[91]
TNAP OE with “wicked high cholesterol” (C57BL/6J-*Ldlr*^Hlb301^/J)	Endothelial cells (*Tie2*-Cre)	Mice displayed increased AP activity in endothelial cells and increased sub-endothelial calcification nodules in their coronary arteries, which recapitulates murine atherosclerosis.	[92,93]
TNAP OE	Endothelial cells (*Cdh5*-Cre)	Over-expression of TNAP on endothelial cells resulted in increased survival and decreased clinical severity post-sepsis compared to controls. Locomotor activity in the last 5 min of open field testing was also increased in VE-cOE mice compared to controls.	[35]
TNAP KO	Osteoblasts and odontoblasts (*Col1a1*-Cre); Early limb bud mesenchyme and a subset of craniofacial mesenchyme (*Prx1*-Cre)	While both Cre recombinase drivers result in similar phenotypes with regards to skeletal defects in cortical and trabecular bone, *Prx1*-cKO mouse long bones appeared more severely affected. Both models resulted in increased osteoclast numbers on alveolar bone surfaces and reduced alveolar bone height. Both models resulted in cementum and periodontal ligament defects, consistent with periodontal disease.	[94]
TNAP KO	Endothelial cells (*Cdh5*-Cre)	Primary BMECs revealed decreased barrier integrity compared to controls, which was mitigated after treatment with fasudil.	[37] (results reported in a preprint)

Key: OE = overexpression, WT = wild-type, KO = knock-out.
